# Treatment of ankylosing spondylitis complicated with a thoracolumbar Andersson lesion by posterior closed osteotomy, debridement and fusion through the fracture line

**DOI:** 10.1186/s12891-022-05770-3

**Published:** 2022-08-26

**Authors:** Chaofeng Guo, Tao Li, Hongqi Zhang, Qile Gao, Gengming Zhang, Jinyang Liu, Yuxiang Wang, Ang Deng, Shaohua Liu, Yang Sun, Mingxing Tang

**Affiliations:** 1 Department of Spine Surgery and Orthopaedics, Xiangya Hospital, Central South University, Changsha, 410008 China; 2 National Clinical Research Center for Geriatric Disorders, Xiangya Hospital, Central South University, Changsha, China

**Keywords:** Andersson lesion (AL), Ankylosing spondylitis, Spinal pseudarthrosis (SP), Kyphosis, Posterior closed osteotomy, debridement and fusion through the fracture line

## Abstract

**Background:**

An Andersson lesion (AL) is a fatigue fracture occurring across three columns in ankylosing spondylitis (AS), resulting in spinal pseudarthrosis (SP) formation, most commonly in the thoracolumbar segment. However, there is still great controversy and few reports on the best surgical method for the treatment of AS combined with thoracolumbar AL. The purpose of this study was to investigate the efficacy of posterior closed osteotomy, debridement and fusion through the fracture line for the treatment of this disease.

**Methods:**

The clinical data of 13 patients (male 8, female 5, mean age 50.6 years) with AS combined with thoracolumbar AL treated with posterior closed osteotomy, debridement and fusion through the fracture line were retrospectively analysed. The following parameters of the full-length lateral spine radiographs were measured preoperatively and at the last follow-up: cervical 7 tilt (C_7_T), global kyphosis (GK), thoracic kyphosis (TK), thoracolumbar kyphosis (TLK), local kyphosis (LK), angle of the fusion levels (AFL), lumbar lordosis (LL), pelvic incidence (PI), pelvic tilt (PT), sacral slope (SS) and sagittal vertical axis (SVA). The visual analog scale (VAS), Oswestry disability index (ODI) and Scoliosis Research Society-22 (SRS-22) scores were recorded preoperatively and at the last follow-up.

**Results:**

The mean operation time was 345 min, the mean blood loss was 673 mL, and the mean follow-up time was 21.9 months. Compared with the preoperative values, the C_7_T, GK, TK, TLK, LK, AFL, PT, SS and SVA values of all patients were significantly improved at the last follow-up (*P* < 0.05); GK improved from 81.62 ± 16.11 to 50.15 ± 8.55, with an average of 31° of correction (F = 75.945, *P*<0.001). The VAS, ODI and SRS-22 scores also significantly improved (*P* < 0.05). At the last follow-up, bone fusion was found in all fracture ends. One patient developed numbness in the lower limbs after surgery and recovered after 3 months of rehabilitation; none of the remaining patients experienced postoperative complications.

**Conclusions:**

Posterior closed osteotomy, debridement and fusion through the fracture line completely removes the necrotic tissue around the SP, relieves symptoms, and corrects kyphosis simultaneously. It reduces the tension behind the fracture line or changes the tension into compressive stress, enabling stable repair of the fracture and avoiding anterior surgery. It is a safe and effective operation.

**Supplementary Information:**

The online version contains supplementary material available at 10.1186/s12891-022-05770-3.

## Background

An Andersson lesion (AL) is a kind of fatigue fracture that occurs in the intervertebral space or vertebral body when ankylosing spondylitis (AS) is not effectively treated. It is also called spinal pseudarthrosis (SP), stress fracture or fatigue fracture [1–3]. Park et al. [4] reported that AL can be divided into inflammatory AL and traumatic AL. Inflammatory AL is the natural progression of AS and conservative treatment is effective, while traumatic AL is the true SP and requires surgical treatment. SP is an insidious fracture line that extends through the disc or vertebral body to the posterior column, allowing abnormal movement. Continuous excessive mechanical tension and anterior component movement lead to further bone resorption and hardening reactions, producing extensive destructive changes [5]. SP in AL patients most commonly occurs at the stress concentrated thoracolumbar junction, and is a kind of nonunion of a three-column fracture [6], which often causes obvious lumbar back pain, sagittal plane imbalance, and even neurological dysfunction [7]. Currently, fixed fusion surgery is considered the preferred treatment for AL patients who have failed conservative treatment [6, 8, 9], with the aim of restoring sagittal balance, relieving pain, promoting fracture healing, and even improving patient survival [10, 11]. However, the pathogenesis and clinical characteristics of this disease are different from those of conventional spinal fractures, and it is difficult to treat and often requires surgical treatment [12]. The surgical methods reported in the literature include posterior fusion [1, 6], anterior fusion [13] and combined anterior and posterior approaches [14]. However, there is still a great deal of debate about the best surgical approach. In this study, the clinical data of 13 patients with AS combined with thoracolumbar AL were retrospectively analysed to investigate the efficacy of posterior closed osteotomy, debridement and fusion through the fracture line for AS combined with thoracolumbar AL.

## Methods

This study was approved by the Ethics Committee of Xiangya Hospital, Central South University, and all patients signed informed consent forms. A retrospective analysis of the clinical data of 13 patients with AS combined with thoracolumbar AL treated with posterior closed osteotomy, debridement and fusion through the fracture line in our hospital from January 2015 to January 2021, including 8 males and 5 females was performed (Table 1). The average age was 50.6 years. The inclusion criteria were as follows: 1. Patients diagnosed with AS combined with thoracolumbar AL; 2. Obvious chest and back pain; 3. Complete clinical data; 4. No significant relief of symptoms after conservative treatment. The exclusion criteria were as follows: 1. Other types of spinal fracture, such as a Chance fracture; 2. Spinal tumours.

**Table 1:** Clinical characteristics of patients

NO	Sex	Age (Years)	Course of disease (Years)	Trauma history	Pseudarthrosis location	Complications
1	F	36	5	No	T12/L1	No
2	F	30	7	No	T10/11	No
3	M	45	20	Yes	L3/4	No
4	M	57	30	Yes	T11/12	Yes
5	M	49	8	Yes	T7	No
6	F	48	20	No	T11/12	No
7	F	51	10	No	T11/12	No
8	M	80	15	Yes	T12	No
9	M	52	7	No	T10/11	No
10	M	58	9	No	T11/12	No
11	F	53	13	No	T10/11	No
12	M	51	16	No	T11/12	No
13	M	48	10	No	T12/L1	No

### Surgical method

After the application of general anaesthesia, the patient was placed in the prone position, and a posterior midline longitudinal incision was made from the spinous process of the fixed segment identified preoperatively. After exposure, 2–3 pairs of pedicle screws were inserted above and below the fracture. First, the laminar fracture space was carefully separated, and subperiosteal dissection was performed along both sides of the spine at the fracture plane, exposing the lateral spine until the spine turned forward, with a range of approximately 2 cm. Then, the lamina intervertebral joint was removed layer by layer at the lamina fracture (the range of removal was determined by the length of posterior column closure required for correction of kyphosis; the larger the kyphosis orthopaedic angle, the greater the amount of lamina that needed to be removed), and the intraspinal dural capsule and corresponding nerve roots were exposed (intraoperative dural separation should be performed carefully due to the lack of epidural adipose tissue in the injured plane). The ventral dural sac was carefully separated from the posterior wall of the spinal canal, and the venous plexus was cauterized with bipolar electrocoagulation to stop the bleeding. A temporary fixed rod was installed on one side, and the satisfactory separation of the dural sac and nerve root on the opposite side were moderately retracted to the midline with the nerve retracer to expose the fracture seam of the anterior wall of the spinal canal and remove the necrotic tissue in the fracture seam. If the kyphosis needed to be corrected at the same time, an ultrasonic bone knife was used to repair the vertebral bone in a backwards v-shaped opening above and below the fracture seam. The same method was used to repeat the operation on the opposite side so that the osteotomy gap on both sides was completely penetrated. After the osteotomy was completed, bone particles or titanium cages filled with bone particles were implanted according to the extent of the bone defect. The temporary fixation rods were removed, and then two fixed titanium rods were cut and prebent according to the normal physiological curvature. The osteotomy gap was gradually closed by holding both sides of the upper and lower screws at the osteotomy site, two rods were placed, and the screws were locked to complete the orthosis. There was no obvious compression of the dural sac in the spinal canal, and the dural sac pulsated. After the wound was rinsed, the lamina and facet joints between the roughened vertebral segments were implanted with autologous or allogeneic bone and placed in transverse joints. One or two drainage tubes were placed according to the wound size, and the wound was sutured layer by layer. Somatosensory evoked potentials and motor evoked potentials were monitored continuously during the operation. All patients wore braces for 3–6 months after surgery and were followed up regularly.

### Radiographic measurements

All patients underwent full-length anteroposterior and lateral spinal radiographs, computed tomography (CT) three-dimensional reconstruction, and magnetic resonance imaging (MRI) before surgery. The following parameters were measured on full-length lateral radiographs of the spine before surgery and at the last follow-up: cervical 7 tilt (C_7_T), global kyphosis (GK), thoracic kyphosis (TK), thoracolumbar kyphosis (TLK), local kyphosis (LK), angle of the fusion levels (AFL), lumbar lordosis (LL), pelvic incidence (PI), pelvic tilt (PT), sacral slope (SS) and sagittal vertical axis (SVA).

### Evaluation of quality of life

Scoliosis Research Society-22 (SRS-22) is also a valid and reliable scale for AS patients [15, 16]. The visual analog scale (VAS), Oswestry Disability Index (ODI) and SRS-22 questionnaires were completed by all patients before surgery and at the last follow-up. The SRS-22 questionnaire included five areas: pain, function, self-image, mental health and satisfaction.

### Statistical analyses

Measurement data are represented as the mean ± standard deviation. A repeated measurement design was used to compare the preoperative and last follow-up data, and post hoc analysis was performed using LSD-t test. Statistical analyses were performed with SPSS 25.0 statistical software (IBM, USA). *P* < 0.05 was regarded as a significant difference.

## Results

### Clinical features of the patients

Thirteen patients (8 males and 5 females) underwent the procedure and were followed up. The mean age of the patients was 50.6 years, and the mean course of disease was 13.1 years (range: 5–30 years). Four of the patients had a history of trauma (Table 1). The mean operation time was 345 min (range: 200–550 min), the mean blood loss was 673 mL (range: 300–1600 ml), and the mean follow-up was 21.9 months (range: 12–36 months).

### Radiologic results of the patients

At the last follow-up, the C_7_T, GK, TK, TLK, LK, AFL, PT, SS and SVA values of all patients were significantly improved compared with those before surgery (*P* < 0.05), and the LL and PI values were not significantly different from those before surgery (*P* > 0.05) (Table 2). At the last follow-up, GK improved from 81.62 ± 16.11 to 50.15 ± 8.55, with an average of 31° of correction (F = 75.945, *P*<0.001, Table 2). LK improved from 21.54 ± 17.73 to 6.38 ± 6.59, with an average of 15° of correction (F = 13.500, *P* = 0.003, Table 2). SVA improved from 83.89 ± 71.22 preoperatively to 45.88 ± 45.23 (F = 10.125, *P* = 0.008, Table 2).

**Table 2:** Comparison of imaging sagittal parameters between the preoperative and last follow-up visits

Sagittal parameters	Preoperative	Last follow-up	*F value*	*P* value
C_7_T (°)	80.45 ± 10.77	86.95 ± 4.92	9.336	0.010*
GK (°)	81.62 ± 16.11	50.15 ± 8.55	75.945	<0.001*
TK (°)	52.15 ± 20.66	39.08 ± 9.21	8.221	0.014*
TLK (°)	38.08 ± 16.61	20.77 ± 9.61	14.545	0.002*
LK (°)	21.54 ± 17.73	6.38 ± 6.59	13.500	0.003*
AFL (°)	37.08 ± 21.34	16.00 ± 11.11	26.162	<0.001*
LL (°)	− 35.46 ± 17.35	− 37.77 ± 14.27	0.562	0.468
PI (°)	47.23 ± 15.93	47.66 ± 10.77	0.016	0.903
PT (°)	29.62 ± 13.19	21.20 ± 9.14	11.847	0.005*
SS (°)	17.62 ± 10.03	26.46 ± 11.34	17.752	0.001*
SVA (mm)	83.89 ± 71.22	45.88 ± 45.23	10.125	0.008*

*P < 0.05

**Fig. 1:**
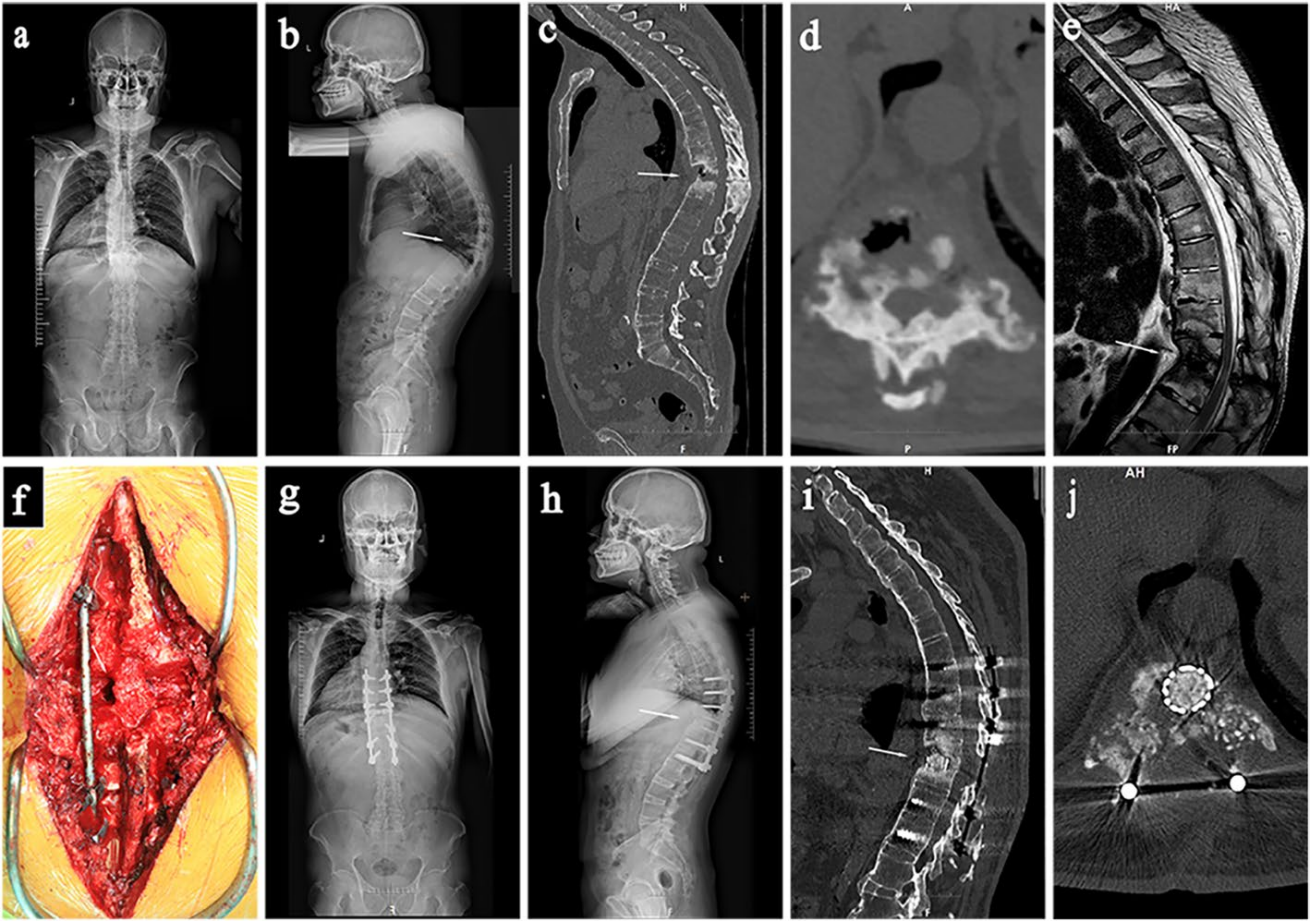
A 52-year-old male patient, diagnosed with AS combined with T10/11 AL. **a** Preoperative full length anteroposterior radiograph of spine. **b** Preoperative full length lateral radiograph of spine showed excessive thoracic kyphosis and a T10/11 intervertebral fracture line. **c**, **d** Preoperative CT sagittal view and cross section showed T10/11 intervertebral space involving a three-column fracture and a visible vacuum sign. **e** Preoperative MRI showed a low signal on T10/11 intervertebral space T2WI; **f** The osteotomy gap exposed through the fracture line during the operation. **g** Full length anteroposterior radiographs of the spine 18 months after surgery. **h** Full-length lateral spinal radiographs 18 months after surgery showed normal thoracic kyphosis, and a titanium cage was inserted into the T10/11 intervertebral space. **i**, **j** Sagittal and cross - sectional CT images at 18 months postoperatively showed intervertebral fusion with the bone graft

**Fig. 2:**
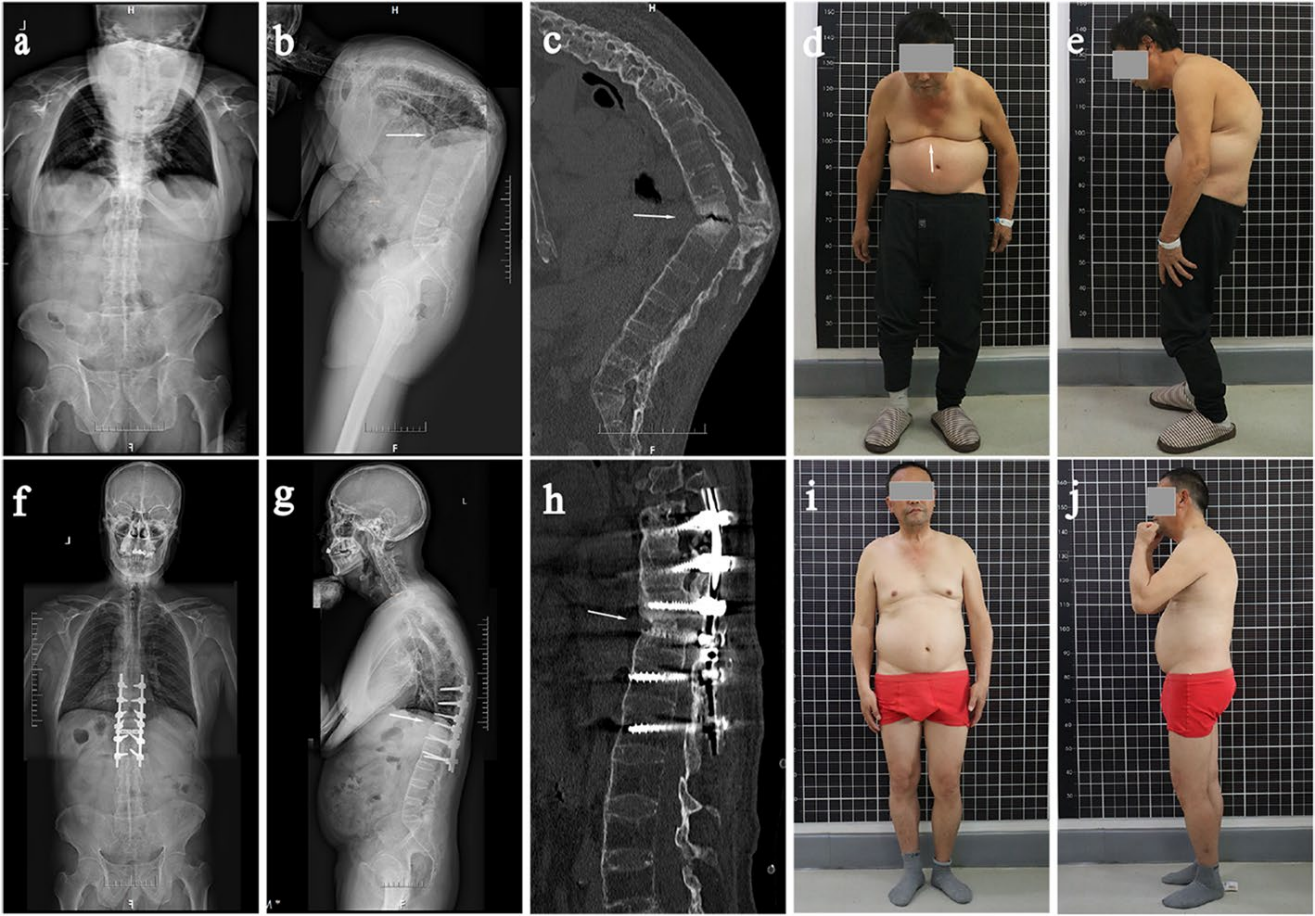
A 58-year-old male patient, diagnosed with AS combined with T11/12 AL. **a** Preoperative full length anteroposterior radiograph of spine. **b** Preoperative lateral spinal radiographs showed excessive thoracic kyphosis and a T11/12 intervertebral fracture line (white arrow). **c** Preoperative CT sagittal view showed T11/12 intervertebral space involving a three-column fracture and a visible vacuum sign (white arrow). **d**, **e** Preoperative gross image showing severe kyphosis of the spine with abdominal wall wrinkles (white arrow); **f** Full length anteroposterior radiographs of the spine 12 months after surgery. **g** Full length lateral spinal radiographs 12 months after surgery showed normal thoracic kyphosis. **h** Sagittal CT images at 12 months postoperatively showed intervertebral fusion with the bone graft. **i**, **j** Postoperative gross radiography showed normal physiological curvature of the thoracolumbar spine, and the appearance was significantly improved

At the last follow-up, bone fusion was obtained at all fracture ends, without internal fixation loosening, prolapse, fracture, etc., and without significant correction angle loss or SP recurrence. Typical cases are shown in Figs. 1 and 2.

### Evaluation of quality of life

The VAS, ODI and SRS-22 scores of all patients at the last follow-up were significantly improved compared with those before surgery (*P* < 0.05, Table 3). The VAS score improved from 7.77 ± 0.83 to 2.62 ± 1.61, which was statistically significant (F = 161.281, *P*<0.001, Table 3). The ODI score significantly improved from 87.85 ± 6.84 to 37.78 ± 21.08 (F = 71.721, *P*<0.001, Table 3). The SRS-22 scores of all patients at the last follow-up were significantly improved in five areas: pain, function, self-image, mental health and satisfaction compared with the preoperative scores (Table 3). One patient developed numbness in the lower limbs after surgery and recovered after 3 months of rehabilitation. At the last follow-up, all patients were satisfied with the results of the surgery.

**Table 3:** Comparison of the VAS, ODI and SRS-22 scores between the preoperative and last follow-up visits

	Preoperative	Last follow-up	*F value*	*P* value
VAS	7.77 ± 0.83	2.62 ± 1.61	161.281	<0.001*
ODI (%)	87.85 ± 6.84	37.78 ± 21.08	71.721	<0.001*
SRS-22				
Pain	2.69 ± 0.48	4.54 ± 0.52	93.405	<0.001*
Function	2.85 ± 0.80	4.46 ± 0.52	80.182	<0.001*
Self-image	2.54 ± 0.52	4.46 ± 0.52	117.187	<0.001*
Mental health	3.00 ± 0.71	4.46 ± 0.52	46.085	<0.001*
Satisfaction	2.54 ± 0.52	4.69 ± 0.48	196.000	<0.001*

*P < 0.05

## Discussion

At present, the aetiology of SP in AS is not clear and may involve a variety of mechanisms. Most scholars believe that SP is caused by mechanical stress changes based on the destruction of bone by the inflammatory reaction involved in AS [12, 17]. As stress increases, patients with advanced AS may develop fatigue fractures with minor or no trauma at all [18]. Fatigue fractures are most likely to occur in the thoracolumbar segment (T11 - L1) with high stress concentrations, and fracture lines are more common in intervertebral spaces with low resistance [1, 13]. In our study, all patients had traumatic AL, which failed to respond to conservative treatment, and the presence of SP was confirmed by imaging, of which 62% had fracture lines located in this region, 85% had fracture lines passing through the intervertebral space, and only 31% had a history of trauma (Table 1), indicating that mechanical factors play an important role in SP formation in AL patients.

SP in AL patients is a nonunion state that crosses the three columns of the spine, and the compensatory hyperplasia of osteophytes and fibrotic tissues around it often fail to heal the fracture (Figs. 1c, 2c). In addition, these osteophytes and fibrotic tissues may compress the dural sac or nerve roots to varying degrees, causing severe pain and neurological symptoms [14]. Therefore, these fractures are different from conventional fractures, require more fusion and stability, and often require surgery. The best procedure for these patients is still highly controversial. In the past, the following three surgical methods were used to treat AL: 1. Anterior approach: Fang et al. [13] believed that the anterior approach enabled direct removal of the lesions and facilitated bone grafting, making it the best surgical method for SP treatment. However, there are many postoperative complications, such as screw loosening and protrusion, which cannot completely correct the kyphosis [1, 13, 19]. 2. Combined anterior and posterior surgery: Kim et al. [14] performed a Smith–Petersen osteotomy (SPO) at the same level for 12 AL patients to correct the sagittal deformity and then performed anterior surgery to repair the fatigue fracture. Although the correction of kyphosis and promotion of bone union was achieved, anterior and posterior surgeries involve a large amount of trauma, a large amount of blood loss and a high risk of complications. 3. Posterior surgery: Chang et al. [1] reviewed and analysed 30 AL patients who underwent posterior osteotomy and orthopaedic internal fixation alone, which corrected LK of 38°on average. It is a safe and effective method for the treatment of AL and does not involve additional anterior fusion, but it is only applicable to patients with less obvious anterior damage [1]. Shaik et al. [20] performed posterior long segment in situ spinal fusion using the pedicle screw system for 18 AL patients without anterior bone grafting or posterior osteotomy, believing that posterior approach alone, long segment fixation and posterior spinal fusion is a safe, simple and rapid method for the treatment of AL patients and can prevent the morbidity of anterior surgery. However, the study only measured the reduction of kyphosis based on clinical and radiological features and did not perform a detailed quantitative analysis of deformity correction. Therefore, the curative effect of this operation needs to be further studied. Wang et al. [6] followed up 12 patients with thoracolumbar AL who underwent posterior surgery and found that solid bony fusion was achieved in all cases with no corrective loss during follow-up, so this option avoided anterior surgery. They used a single posterior approach through the foramina or pedicle where SP was located to remove the SP tissue and perform bone grafting after complete debridement. At the same time, they could also perform spinal decompression for patients with neurological deficits and orthopedics for patients with spinal malformations. In this study, 13 AS patients with thoracolumbar AL were treated with the posterior closed osteotomy, debridement and fusion through the fracture line approach, and the SP was firmly fixed and fused, which significantly improved pain symptoms and nerve function (Table 3) and achieved satisfactory orthopaedic effects (Table 2). The surgical method in this study was similar to that reported by Wang et al. We also scraped away the anterior lesion tissue and performed bone grafting. However, for patients with large bone defects, we placed titanium mesh filled with bone particles, which increased the chance of bone contact, strengthened the anti-buckling stress effect of internal fixation, reduced intervertebral stress, and better promoted anterior middle column healing. This method can promote fracture healing through the following three main factors: 1. The posterior column on the tension side is fixed by inserting pedicle screws to reduce the tension at the fracture end or change the tension to compressive stress: the internal fixator is relatively stable on the tension side of the fracture and has good control force, which can reduce the tension or change the tension to compressive stress, in line with the biomechanical properties of the spine, and achieve firm fixation of the fracture. 2. The SP was excised, and necrotic tissue was completely removed through the fracture gap. Through natural fracture gap resection SP, the step of artificial osteotomy is omitted, and the surgical complications are reduced. The necrotic tissue around the SP was completely removed through the fracture gap, which not only relieved the patient’s pain symptoms but also provided a good wound surface for fracture healing. 3. Narrowing fracture gap: there are still osteotomy gaps associated with the fracture osteotomy, and titanium mesh is placed to implant autologous bone or allogeneic bone according to the range of bone defects, which can promote fracture healing.

In addition, there is currently no accepted standard for fixation of AS with a thoracolumbar AL. Reinhold et al. [21] recommended that AS combined with thoracolumbar AL patients be treated with posterior internal fixation extending 2 to 3 levels above and below the fracture segment to maintain adequate reduction and stability until the fracture healed. At present, many scholars believe that AS patients with thoracolumbar AL require fixation of at least two segments above and below the fracture [1, 22]. All 13 AL patients in this study had at least 2 segments fixed above and below the lesion, and all achieved satisfactory therapeutic results. However, the selection of specific surgical methods and fixed segments for AS combined with thoracolumbar AL patients still needs to be made according to the actual situation of the patients, such as the degree of kyphosis, the degree of ossification of the affected vertebra, and the degree of intervertebral stenosis. The subjects included in this study and the follow-up time were limited, and the sample size should be increased for a longer follow-up study in the later stage.

## Conclusions

In the treatment of AS combined with thoracolumbar AL patients, the posterior closed osteotomy, debridement and fusion through the fracture line can be used to completely remove the necrotic tissue around the SP and remove the SP. Meanwhile, the tension behind the fracture line can be reduced or changed to compressive stress to narrow the fracture gap and achieve solid fitting and fixation of the fracture end. In addition, for the patients with obvious kyphosis, simultaneous orthosis can improve the appearance with fewer complications, and it is a safe and effective surgical procedure.

## Supplementary Information

**Additional file 1: Supplementary Table 1.** Basic information and original data of 13 patients.

## Data Availability

All data generated or analysed during this study are included in this published article [and its supplementary information files].

## Abbreviations

AL: Andersson lesion
AS: Ankylosing spondylitis
SP: Spinal pseudarthrosis
C_7_T: Cervical 7 tilt
GK: Global kyphosis
TK: Thoracic kyphosis
TLK: Thoracolumbar kyphosis
LK: Local kyphosis
AFL: Angle of the fusion levels
LL: Lumbar lordosis
PI: Pelvic incidence
PT: Pelvic tilt
SS: Sacral slope
SVA: Sagittal vertical axis
VAS: Visual analog scale
ODI: Oswestry disability index
SRS-22: Scoliosis Research Society-22
CT: Computed tomography
MRI: Magnetic resonance imaging
SPO: Smith–Petersen osteotomy
